# Cat-Scratch Disease in the United States, 2005–2013

**DOI:** 10.3201/eid2210.160115

**Published:** 2016-10

**Authors:** Christina A. Nelson, Shubhayu Saha, Paul S. Mead

**Affiliations:** Centers for Disease Control and Prevention, Fort Collins, Colorado, USA (C.A. Nelson, P.S. Mead);; Emory University, Atlanta, Georgia, USA (S. Saha)

**Keywords:** Cat scratch disease, *Bartonella henselae*, lymphadenopathy, zoonotic disease, fleas, zoonoses, bacteria, United States

## Abstract

Cat-scratch disease (CSD) is mostly preventable. More information about the epidemiology and extent of CSD would help direct prevention efforts to those at highest risk. To gain such information, we reviewed the 2005–2013 MarketScan national health insurance claims databases and identified patients <65 years of age with an inpatient admission or outpatient visit that included a CSD code from the International Classification of Diseases, Ninth Revision, Clinical Modification. Incidence of CSD was highest among those who lived in the southern United States (6.4 cases/100,000 population) and among children 5–9 years of age (9.4 cases/100,000 population). Inpatients were significantly more likely than outpatients to be male and 50–64 years of age. We estimate that each year, 12,000 outpatients are given a CSD diagnosis and 500 inpatients are hospitalized for CSD. Prevention measures (e.g., flea control for cats) are particularly helpful in southern states and in households with children.

## Abstract 2

Each year, this preventable disease affects about 12,500 persons, mostly those who live in the South and are 5–9 years of age.

Cat scratch disease (CSD) is a zoonosis caused by *Bartonella henselae,* a fastidious, hemotropic, gram-negative bacterium. *B. henselae* is maintained and spread among cats—the principal mammal reservoir species—by the cat flea (*Ctenocephalides felis*); transmission to humans occurs via scratches, and possibly bites, from cats. CSD occurs throughout the United States and worldwide wherever cats and their fleas are found (*1*). Knowledge of this emerging pathogen continues to expand; additional *Bartonella* species, such as *B. clarridgeiae*, and mammal hosts, including dogs, have also been linked to CSD (*2*–*4*).

The predominant clinical feature of CSD is lymphadenopathy proximal to the site of a cat scratch or bite; in many patients, a papule develops at the initial wound site before onset of lymphadenopathy. Some patients with *B. henselae* infection experience more serious manifestations, such as neuroretinitis, Parinaud oculoglandular syndrome, osteomyelitis, encephalitis, or endocarditis (*1*). *B. henselae* infection can be particularly severe for patients with immunocompromising conditions, such as AIDS, in whom vascular proliferative lesions (bacillary angiomatosis and bacillary peliosis) may develop (*5*).

In the United States, CSD is not a notifiable condition; therefore, information on the epidemiology of this disease has been limited to clinical case series and analyses of hospital discharge databases. Jackson et al. used records from the Commission on Professional and Hospital Activities (1983–1989) and the National Hospital Discharge Survey (1978–1989) to estimate incidence of CSD hospitalizations to be 0.77–0.86/100,000 population/year. Most hospitalized patients were <18 years of age (55%) and male (60%); highest incidence was in the southern United States. Although the authors also extracted outpatient records from the National Ambulatory Medical Care Survey, only 10 cases of CSD were identified, limiting analysis and extrapolation (*6*).

Reynolds et al. analyzed the Kids’ Inpatient Database for the yeat 2000 and estimated the incidence of CSD hospitalizations to be 0.60/100,000 children <18 years of age/year. In this study, incidence was also highest in southern states but was slightly higher among girls and women (*7*).

Although the syndrome of CSD was first defined in 1950, identification of the etiologic agent and development of diagnostic tests did not occur until the mid-1980s and later (*8*). Therefore, the epidemiology of CSD may have changed in the past few decades because of improved diagnostic tests for CSD and other conditions that mimic CSD (*9*). Patients with typical signs of CSD and compatible exposure history can be given a presumptive clinical diagnosis; diagnostic tests such as serology, PCR, and culture can be useful for confirming typical CSD or for diagnosing atypical CSD (*1*).

To better define the current epidemiology and burden of CSD in the United States, we analyzed a large medical claims database to 1) describe national patterns of clinician-diagnosed CSD among inpatients and outpatients, 2) evaluate changes in disease patterns during the study period, and 3) identify demographic groups at higher risk for CSD. Improved understanding of CSD may facilitate recognition by clinicians and identification of risk groups for whom education about cats and flea control is particularly helpful.

## Methods

We conducted a retrospective analysis of the Truven Health MarketScan Commercial Claims and Encounters database (Truven Health Analytics, Ann Arbor, MI, USA) for 2005–2013. This database contains information on employer-sponsored private health insurance claims from employees, their spouses, and their dependents from all 50 states. The database contains records for persons <65 years of age only.

The MarketScan database includes information about inpatient admissions, outpatient clinic visits, and emergency department visits with associated billing codes from the International Classification of Diseases, Ninth Revision, Clinical Modification (ICD-9-CM). A case was defined as illness in any patient with an insurance claims record for inpatient or outpatient care that included the ICD-9-CM code for cat-scratch disease (078.3) as either a principal or secondary diagnosis. Only the first record with the 078.3 code was counted and considered the incident event. Patients who had been admitted as inpatients and had also had outpatient visits were counted as inpatients.

Because some persons in the database were covered by an included health insurance plan for only part of each year, we calculated the denominators for incidence calculations by summing the total number of person-months by year, region, age, and sex. We then divided these sums by 12 to yield the number of person-years for each category. To assess changes in the epidemiology of CSD over time, we divided the dataset into two 3-year periods (2005–2007 and 2011–2013), with a washout period of 3 years in between.

The cost of hospitalization and outpatient visits was calculated by summing total payments for the initial encounter plus any subsequent encounter with the 078.3 code up to 1 year afterward. Total costs included the primary insurance payment, co-insurance, and patient copayment. Costs for procedures such as phlebotomy and laboratory testing were included if they had an associated 078.3 code. Costs of inpatient medications were incorporated into the total payments for hospital stay. Outpatient prescriptions were included in the total cost calculation if they were for an antimicrobial drug recommended for treatment of CSD and prescribed within 30 days before or after the initial outpatient 078.3 encounter (*10*,*11*).

To estimate the total annual number of US patients <65 years of age with a CSD diagnosis, we first calculated age-specific and US census region–specific rates of CSD within the MarketScan database. We then performed direct standardization by multiplying these rates by the US population by age and region. Population estimates were obtained from the 2010 US Census Bureau Population Survey (*12*).

Descriptive statistics and comparisons were performed by using SAS version 9.3 (SAS Institute, Cary, NC, USA). To compare categorical data between inpatients and outpatients and between the periods 2005–2007 and 2011–2013, we calculated risk ratios (RRs).

The protocol underwent human subjects review at the Centers for Disease Control and Prevention and was determined not to be research involving human subjects. As such, institutional review board approval was not required.

## Results

### Study Population

During 2005–2013, the MarketScan database contained information on a median of 39,970,145 enrollees for each year (range 16,159,068–53,131,420). A total of 280,522,578 person-years were analyzed during the 9-year study period.

### Incidence and Geographic Distribution

During the study period, we identified 13,273 patients with a diagnosis of CSD: 12,735 outpatients and 538 inpatients. Average annual incidence was 4.5 outpatient diagnoses/100,000 population (range 4.0–5.7/100,000) and 0.19 inpatient admissions/100,000 population (range 0.17–0.22/100,000).

**Figure 1:**
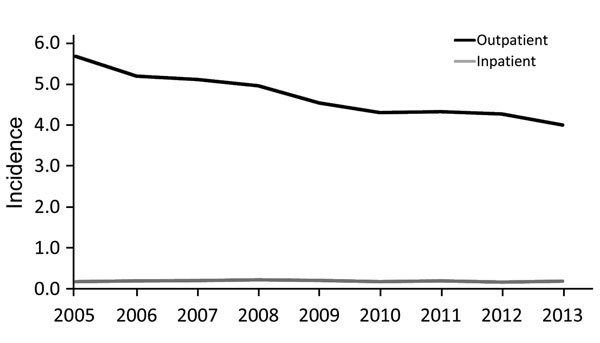
Average annual incidence (cases/100,000 population) of cat-scratch disease outpatient diagnoses and inpatient admissions by year, United States, 2005–2013.

Annual incidence of outpatient CSD diagnoses was highest in 2005 (5.7/100,000 population) then steadily declined to a low of 4.0/100,000 population in 2013 (Figure 1). The decrease in incidence over time occurred primarily in southern states. For inpatient admissions, annual incidence remained relatively stable during the study period and peaked slightly in 2008 (0.22/100,000 population).

**Figure 2:**
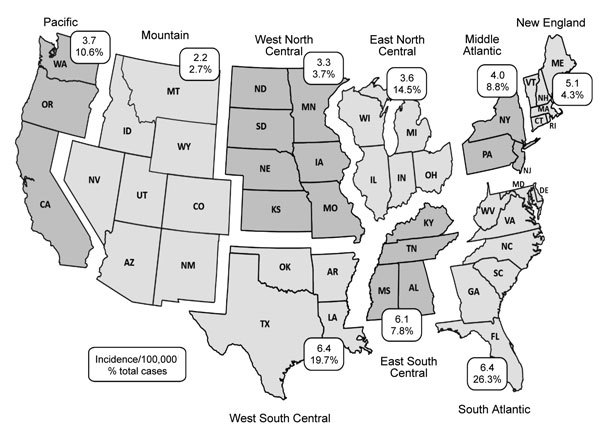
Geographic distribution of cat-scratch disease by US census division, United States, 2005–2013. Rates are reported as average incidence per 100,000 population per year. During the study period, there were <10 cases in Alaska and <10 cases in Hawaii.

Incidence was highest in the West South Central, East South Central, and South Atlantic divisions (6.1–6.4 cases/100,000 population) and lowest in the more arid Mountain division, where cat fleas are less common (2.2 cases/100,000 population) (Figure 2). The highest overall proportion of cases occurred in the South Atlantic division (26.3%), followed by the West South Central division (19.7%).

### Distribution by Age and Sex

**Figure 3:**
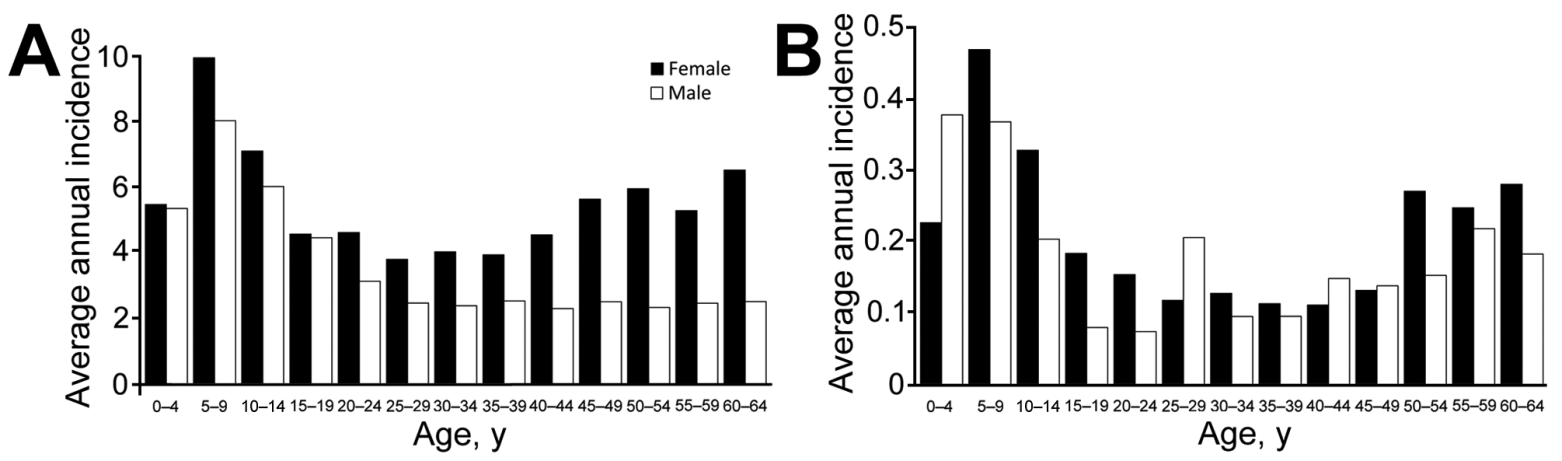
Age- and sex-specific incidence (cases/100,000 population) of cat-scratch disease outpatient diagnoses (A) and inpatient admissions (B), United States, 2005–2013.

Highest average annual CSD incidence for outpatients and inpatients was among children 5–9 years of age (9.0 cases/100,000 patients and 0.4 cases/100,000 patients, respectively) (Figure 3). Children <14 years of age accounted for 32.5% of diagnoses overall. Among adults, highest incidence for outpatients and inpatients was among women 60–64 years of age (6.6 cases/100,000 and 0.3 cases/100,000, respectively) (Figure 3).

Women and girls accounted for 62.0% of outpatient diagnoses and 55.6% of inpatient diagnoses. Although most inpatients were female, inpatients were significantly more likely than outpatients to be male (RR 1.17, 95% CI 1.06–1.29) (Table). Incidence among female patients was higher than that among male patients in all age groups with the notable exceptions of inpatients 0–4, 25–29, and 40–49 years of age. The incidence difference between adult female and male patients widened as age increased.

**Table:** Demographic characteristics of patients with cat-scratch disease, United States, 2005–2013*

Characteristic	Inpatients, no. (%) n = 538	Outpatients, no. (%) n = 12,735	Risk ratio (95% CI)
Male sex	239 (44.4)	4,842 (38.0)	**1.17 (1.06–1.29)**
Age, y			
Child, <14	191 (35.5)	4,128 (32.4)	1.10 (0.97–1.23)
Older adult, 50–64	157 (29.2)	2,971 (23.3)	**1.25 (1.09–1.43)**
Month of diagnosis			
Aug-Nov (late summer and fall)	233 (43.3)	4,764 (37.4)	**1.16 (1.05–1.29)**
Jan	54 (10.0)	1,305 (10.2)	0.98 (0.76–1.27)
Residence in southern state†	314 (58.4)	6,828 (53.6)	**1.09 (1.01–1.17)**
Residence in rural area‡	118 (21.9)	3,056 (24.0)	0.91 (0.78–1.08)

*Boldface indicates statistical significance between comparison groups**.** †Includes states in the South Atlantic, East South Central, and West South Central divisions (Figure 2). ‡Defined as residence of primary beneficiary outside of a Metropolitan Statistical Area.

### Seasonality

**Figure 4:**
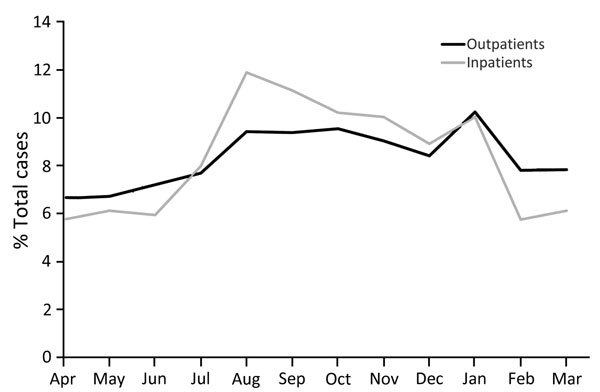
Seasonal variation of cat scratch disease outpatient diagnoses and inpatient admissions, United States, 2005–2013.

**Figure 5:**
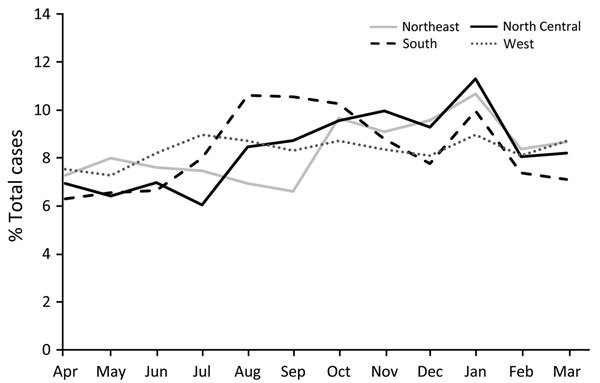
Seasonal variation of cat-scratch disease diagnoses by region, United States, 2005–2013. Northeast region = New England and Middle Atlantic divisions; North Central = East North Central and West North Central; South = South Atlantic, East South Central, and West South Central; West = Mountain and Pacific (divisions shown in Figure 2).

The largest proportion of diagnoses was made during January (10.2%), followed by August–November (9.1%–9.6%/month) (Figure 4). Diagnoses were significantly more likely to be made during August–November for inpatients than for outpatients (Table). Of note, when data were stratified by region, a spike in January was apparent for all regions but most pronounced in the North Central region and least pronounced in the West region (Figure 5).

To account for differences in enrollment by time of year, we also compared incidence by using monthly insurance enrollment figures and incidence among the subset of persons enrolled for the entirety of each year. A similar monthly pattern remained, including the notable spike in January. When stratified by age group, patients 10–49 years of age were most commonly given their diagnosis in January, whereas patients <9 years and >50 years of age were most commonly given their diagnosis during August–November.

### Comparisons over Time

During the 3-year period of 2005–2007, a total of 2,881 patients were given a CSD diagnosis (incidence 5.5 cases/100,000 population/year). During 2011–2013, a total of 5,522 patients received a CSD diagnosis (incidence 4.4 cases/100,000 population/year). The MarketScan database included records from substantially more patients during the latter period, hence the lower overall incidence despite a greater number of patients with CSD.

The proportion of patients with CSD who were hospitalized increased from 3.5% in 2005–2007 to 4.2% in 2011–2013. Patient sex or residence in a rural area did not differ significantly between the 2 periods. Patients with CSD in 2011–2013, as opposed to 2005–2007, were significantly less likely to be <14 years of age (RR 0.79, 95% CI 0.74–0.84), to have been given their diagnosis during August–November (RR 0.92, 95% CI 0.87–0.97), and to be from southern states (RR 0.76, 95% CI 0.73–0.79).

### Estimated Cost and Extrapolation to the US Population

The mean cost of care for outpatients with CSD was $244/patient (median $100; interquartile range [IQR] $68–$169). The mean cost of an inpatient admission plus follow-up care for CSD was $13,663 (median $8,525; IQR $5,535–$15,273). Median length of stay for inpatient admissions was 3 days (IQR 2–5 days).

After directly standardizing age- and region-specific CSD incidence rates to the US population, we estimated that each year 12,000 outpatient diagnoses are made among patients <65 years of age. In addition, each year ≈500 patients <65 years of age are hospitalized for CSD. Using these estimates, we calculated the total annual cost of CSD among persons <65 years of age to be $2,928,000 for outpatients and $6,832,000 for inpatients. Thus, in the United States, the total direct medical costs for CSD are estimated to be $9,760,000/year.

## Discussion

Using data from a large national health insurance claims database, we estimated that the annual incidence of CSD is 4.7 per 100,000 persons <65 years of age and that a total of 12,500 patients in this age group receive a CSD diagnosis each year in the United States. The highest rates of outpatient diagnoses and inpatient admissions for CSD occur among children 5–9 years of age. 

The epidemiology of CSD inpatient admissions described in this study was in some regards similar to that found by previous studies of national hospitalization databases (*6*,*7*). For example, we found that cases occurred predominantly in southern states and during the late summer and fall. Furthermore, incidence of outpatient diagnoses and inpatient admissions in the MarketScan database was highest among children <14 years of age. We also found, however, that children <14 years of age accounted for a smaller proportion of inpatient admissions (35.5%) than that found by the Jackson et al. analysis of hospital databases (45%–50%) (*6*). This difference is surprising given that the MarketScan databases include information on insured persons <65 years of age only, so the proportion of children with CSD in our analysis is artificially inflated.

Although very little data on incidence of CSD in outpatients have been published, our estimate (4.5 cases/100,000 population) is similar to that reported from an outpatient survey conducted in the Washington, DC, metropolitan area (3.3 cases/100,000 population) (*13*). Although Jackson et al. reported a higher estimated incidence (9.3 cases/100,000 population), this extrapolation was based on only 10 patients identified in an ambulatory care database (*6*). The incidence of outpatient diagnoses—but not inpatient admissions— in the MarketScan database clearly and steadily decreased during the study period and should be further evaluated by use of other data sources.

The incidence of inpatient admissions reported in this study (0.19/100,000 population) is substantially lower than that reported in 2 studies of national hospitalization databases (0.60–0.86/100,000). Although Jackson et al. and Reynolds et al. used different data sources, these studies relied on the same basic method of using ICD-9-CM code 078.3 to identify cases. The reported incidences in both previous studies, however, had wider variation and were limited by smaller sample sizes (*6*,*7*). 

The lower incidence of inpatient admissions found by our study is surprising, given that the number of US households with cats has increased in recent decades to an all-time high of 45 million (*14*). The overall proportion of CSD patients who were hospitalized may be artificially low because our analysis did not include patients >65 years of age, who typically have more comorbidities and thus are more likely to be hospitalized. It is also possible that patients with atypical manifestations of CSD were given ICD-9-CM codes for specific clinical conditions (e.g., encephalitis) but not the underlying etiology and so were not included according to our search criteria.

Greater availability and efficacy of flea control products may have reduced risk for *B. hensalae* transmission to humans. Furthermore, while laboratory diagnosis of CSD has improved in recent decades—which may bolster the calculated incidence of CSD—diagnostic capability for other causes of lymphadenopathy (e.g., neoplasms) has also advanced and may have prevented overdiagnosis of CSD. 

It is unclear why an unusually high number of cases occurred in January. Jackson et al. also observed an increase in hospitalizations during January, although the increase was not as striking (*6*). A study of Lyme disease, which used the same MarketScan database, did not show an unusual peak in January, suggesting that it is not artifact from changes in insurance coverage at the beginning of each year (*15*). Kittens are typically born in the spring and adopted during the summer (*16*), and kittens 0–6 months of age have been shown to be at greatest risk for *B. henselae* bacteremia. Conversely, *C. felis* flea abundance peaks during fall and winter, and the highest risk for *B. henselae* bacteremia among cats is during winter (*17*,*18*). These seasonal variations elucidate the reasons for increased incidence of CSD during the fall but do not fully explain the decrease in December and subsequent peak in January.

One hypothesis to explain the January peak is that cats are adopted from shelters more often during the holiday season, for sentimental reasons or as gifts. However, we are unaware of any comprehensive data on cat adoption statistics by month, so assessing this relationship is difficult. Of note, stratified analysis showed that the January peak occurred primarily among patients 10–49 years of age. Another hypothesis is that teenagers and middle-aged adults, who typically spend a great deal of time at school or work, spend more time during the holidays at home in contact with their cats or traveling to other houses with cats. Also, cats spend more time indoors as temperatures decrease during the winter. These and other potential explanations for the unexpected January peak in disease should be explored further.

In contrast to several previous studies that reported an overall predilection of CSD among male patients (*6*,*7*,*19*,*20*), our analysis indicated that only 44.4% of inpatients and 38.0% of outpatients were male. The reasons for this discrepancy are unclear and should be explored further. Compared with outpatients, inpatients were more likely to be male (although a minority of inpatients were male), be 50–64 years of age, and reside in the South. These differences could results from risk factors for severe disease that should be explored further. Differences could also result from more accurate diagnosis for inpatients, rather than true epidemiologic differences.

Our study has several limitations. First, the case definition relies on diagnosis by clinicians and subsequent coding by clinicians or billing specialists, both of which are subject to error. For example, the 078.3 code could have been inappropriately used for care of a cat scratch wound but not actual CSD. Also, in some cases, the 078.3 code may have been recorded as a rule-out diagnosis when CSD was not actually confirmed. To our knowledge, there are no data on the sensitivity and specificity of the 078.3 code for CSD. Furthermore, the MarketScan database is a convenience sample and does not include insurance claims from persons >65 years of age, military personnel, uninsured persons, or Medicaid/Medicare enrollees, among whom risk for CSD may differ. Last, the calculated medical cost of CSD is probably an underestimate because some CSD-related visits and procedures may have been missing the 078.3 code and we did not account for indirect costs such as time away from work.

CSD causes a substantial burden of disease nationwide and disproportionately affects children. Because CSD is a zoonotic infection that is maintained and spread among cats by fleas, comprehensive flea control for cats can help reduce the risk for human infection. Risk may also be reduced by washing hands after contact with cats, to remove potentially infectious flea feces that could enter breaks in the skin. Furthermore, because cats that hunt outdoors are at substantially greater risk for *B. henselae* bacteremia (*17*), limiting hunting activity of cats may reduce risk for human infection. Educational efforts should focus on cat owners, particularly those with children in the household or those with immunocompromising conditions. Additional research is warranted to elucidate the reasons for epidemiologic differences noted in this study and risk factors for severe disease.
